# *getITD* for *FLT3*-ITD-based MRD monitoring in AML

**DOI:** 10.1038/s41375-019-0483-z

**Published:** 2019-05-14

**Authors:** Tamara J. Blätte, Laura K. Schmalbrock, Sabrina Skambraks, Susanne Lux, Sibylle Cocciardi, Anna Dolnik, Hartmut Döhner, Konstanze Döhner, Lars Bullinger

**Affiliations:** 1 Department of Internal Medicine III, University Hospital of Ulm, Ulm, Germany; 2 Department of Hematology, Oncology and Tumor Immunology, Charité University Medicine, Berlin, Germany

**Keywords:** Acute myeloid leukaemia, Cancer genetics

## To the Editor:

The clinical relevance of measurable residual disease (MRD) monitoring has been well recognized in acute myeloid leukemia (AML) [1] and respective assays have been established for several recurrent leukemic markers [2, 3]. However, although internal tandem duplications in the *FLT3* gene (*FLT3*-ITDs) are the most common poor prognosis AML drivers [4], they have remained a challenging target: Their heterogeneity makes conventional PCR-based methods either insensitive or laborious [5]. Yet with recently approved FLT3-kinase inhibitors available [6], a specific monitoring of *FLT3*-mutation loads, and thus response to targeted therapy, is of particular interest. Next-generation sequencing (NGS) workflows for *FLT3*-ITD monitoring have been described, but were previously either proprietary or undisclosed [7, 8], unable to detect and correctly annotate all of the tested ITDs [9, 10], or used in conjunction with manual analysis with inherently subjective results [10].

We have therefore developed a new method based on targeted high-coverage NGS and our novel, open-source analysis program *getITD*. For assay assessment, we sequenced 3 human AML cell lines (Leibniz Institute DSMZ-German Collection of Microorganisms and Cell Cultures, Braunschweig, Germany), 2 healthy volunteers, and 57 samples from 28 AML patients who were all included in the AMLSG BiO Registry study (NCT01252485) and gave their informed consent according to the Declaration of Helsinki. We show that our workflow detects ITDs of a broad range of lengths, insertion sites and variant allele frequencies (VAFs) with high accuracy and precision, is fully objective without any requirement for manual analysis, and thus applicable to routine clinical monitoring. Sample and method details are provided as supplementary information. *getITD* is freely available at https://github.com/tjblaette/getitd.

**Fig. 1:**
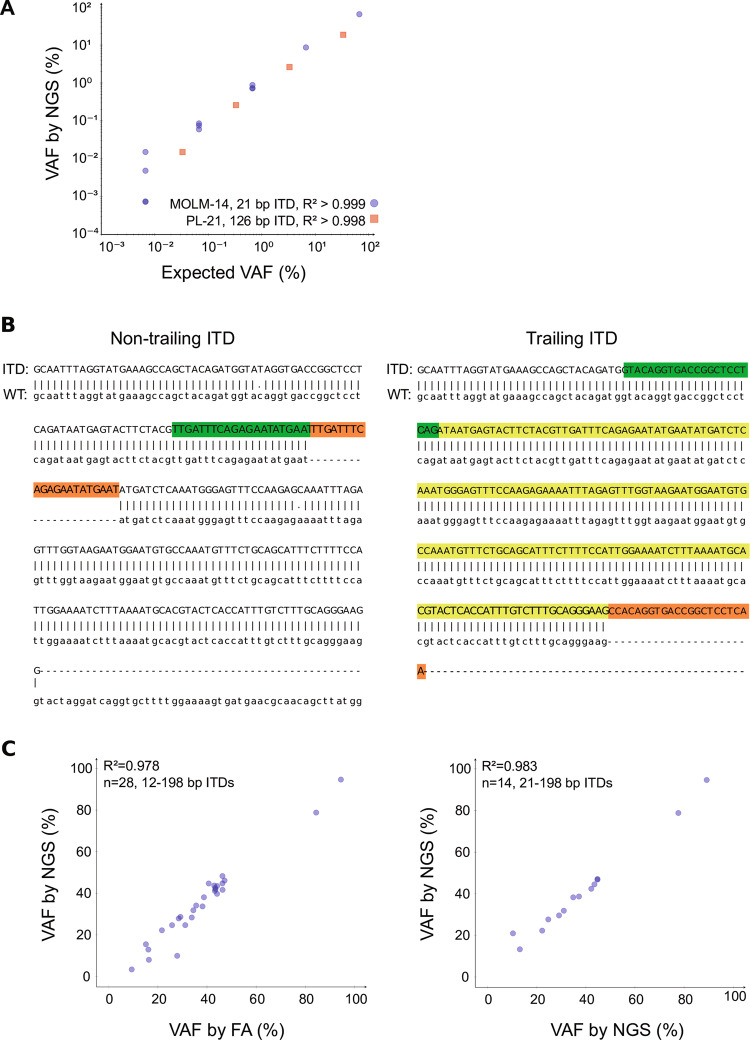
Assessment of assay sensitivity, accuracy, and reproducibility. **a** DNA of *FLT3* internal tandem duplication (*FLT3*-ITD) positive acute myeloid leukemia (AML) cell lines MOLM-14 and PL-21 was each serially diluted in DNA of the *FLT3*-ITD negative AML cell line HL-60; replicates were diluted independently. The expected ITDs were detected in all samples, down to a variant allele frequency (VAF) of 6.7 × 10^−5^. Exact numbers and statistics are presented in the supplementary table S1. **b** Exemplary alignments created by *getITD*, showing the two types of ITDs detected by our assay: a non-trailing 21 bp ITD (left) and a trailing 198 bp ITD (right). ITD inserts are highlighted in red; the respective wild-type (WT) tandems are highlighted in green. For the trailing ITD, a lighter green mark presumably duplicated WT sequence that was not actually sequenced. Symbols “|” and “.” connect matching and mismatching bases, respectively; gaps indicating insertions and deletions are annotated with “-”. **c** Comparison of VAF estimates from PCR- and capillary electrophoresis-based fragment analysis (FA) and our next-generation sequencing (NGS)-based assay for 28 *FLT3*-ITD positive diagnostic AML samples (left) and two independent analyses of 14/28 *FLT3*-ITD positive diagnostic AML patient samples (right). Allelic ratios (ARs) obtained by FA were converted to VAFs for this comparison as described in the supplement

To demonstrate our assay’s specificity, we analyzed three *FLT3*-ITD negative control samples (peripheral blood of healthy volunteers, *n* = 2; AML cell line HL-60). No ITDs were reported, indicating an assay specificity of 100% (coverage: 1.1–4.2 million, mean 2.6 million paired-end reads). To assess sensitivity, we analyzed two serial dilutions of *FLT3*-ITD positive in *FLT3*-ITD-negative DNA from AML cell lines MOLM-14 (21 bp ITD, 67% VAF [11]), PL-21 (126 bp ITD, 33% VAF [11]), and HL-60 (*FLT3*-ITD negative [11]). For each of the ITD-positive cell lines, we sequenced undiluted DNA and 3–4 serial 1:10 dilutions in HL-60 (1.1–2.9 million, mean 2.0 million paired-end reads). The expected ITDs were detected in all samples and VAF estimates were accurate and decreased linearly as expected (MOLM-14: *R*^2^ > 0.999; PL-21: *R*^2^ > 0.998; Fig. 1a). The most diluted sample, the 1:10,000 MOLM-14:HL-60 dilution, harbored the ITD at 0.0067% VAF (6.7 × 10^−5^), demonstrating that our limit of detection is at least this low. Note that while MOLM-14’s 21 bp ITD was correctly identified as such, PL-21’s 126 bp ITD was estimated to be 125 bp long. PL-21’s ITD is too long for the insert and its wild-type (WT) tandem to be spanned by the same read (2 × 126 bp > 250 bp) and, for these *trailing* ITDs, length estimates are non-exact but should deviate by ≤3 bp (Fig. 1b).

To determine assay accuracy, we sequenced the diagnostic *FLT3*-ITD positive bone marrow samples of 28 AML patients (0.04–2.6 million, mean 1.4 million paired-end reads) and compared results to those of previously obtained fragment and Sanger sequencing analyses. In these 28 samples, PCR- and capillary electrophoresis-based fragment analysis (FA) had identified a total of 34 ITDs (1–2 per sample, mean 1.2). All 34 ITDs were also detected by our NGS-based assay. Insertion sites identified by our assay were all identical to those identified by Sanger sequencing and VAF estimates were highly correlated with those from FA (*R*^2^ = 0.978; Fig. 1c). Estimated ITD lengths were identical to those previously determined for 33/34 ITDs; the 198 bp ITD was reported as 197 bp and *trailing*.

In 19/28 samples, our assay identified one or more additional ITDs not detected by FA. In total, our assay detected 105 ITDs in these 28 samples (1–16 per sample, mean 3.8). The 71/105 ITDs, detected by our assay but not conventional FA, were similar in length to those detected by both assays (both: 12–198 bp, mean 50.4 bp; *getITD* only: 9–194 bp, mean 51.6 bp) but present at lower VAFs and thus mostly below the detection limit of conventional methods (both: 9.3–94.5%, mean 38.2%; *getITD* only: 0.006–2.7%, mean 0.2%). To ensure assay reproducibility, we independently re-sequenced 14/28 samples at low coverage (0.2–1.3 million, mean 0.5 million paired-end reads). Results were entirely reproducible between these low- and the above-mentioned high-coverage replicates, with identical insertion sites and lengths and again highly correlated VAFs (*R*^2^ = 0.986; Fig. 1c).

**Fig. 2:**
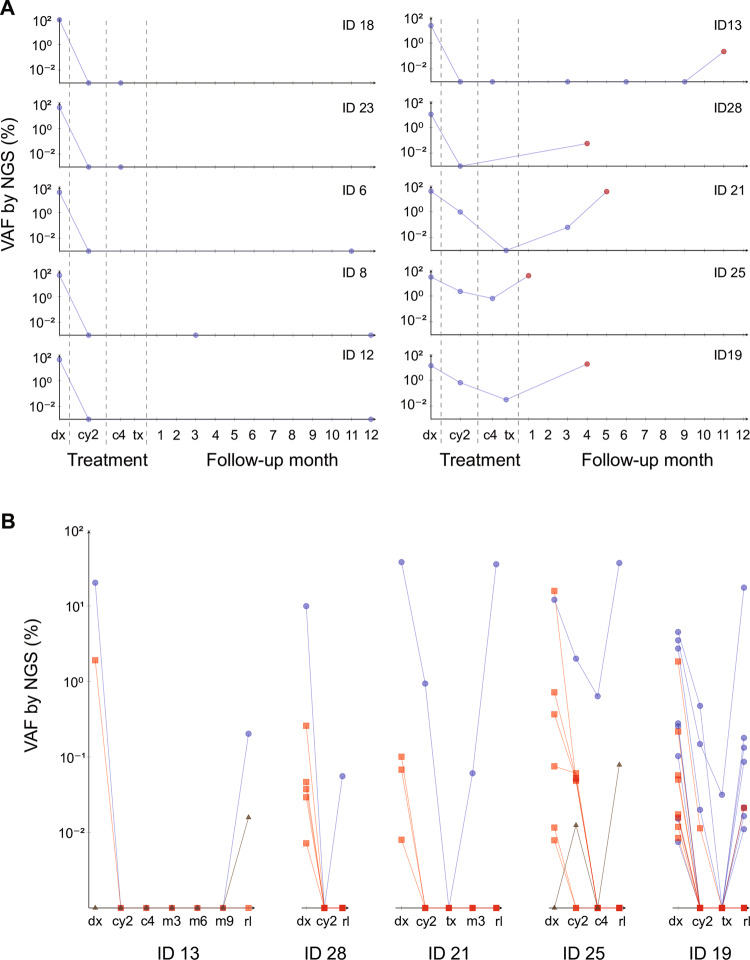
Measurable residual disease (MRD) analysis in acute myeloid leukemia (AML) samples. **a** Serial MRD analysis of five *FLT3* internal tandem duplication (*FLT3*-ITD) positive AML patients who remained in continuous clinical remission over the course of treatment (left) and five *FLT3*-ITD-positive AML patients who relapsed during follow-up (right). Each plot shows the results of a single patient: sequenced samples are marked and connected for better visibility; where applicable the relapse sample is marked in red. Marks on the *x*-axis represent samples which were analyzed but which were *FLT3*-ITD negative by our assay. When multiple ITD clones were detected at a certain time point, their variant allele frequencies (VAFs) were summed for plotting. **b** Serial MRD analysis of individual *FLT3*-ITD clones in the five *FLT3*-ITD positive AML patients who relapsed during follow-up. We analyzed the diagnosis and two to six follow-up samples, including the respective relapse sample, and plotted the change of each clone’s VAF over the sampled time points. Clones persisting from diagnosis (dx) to relapse (rl) are shown as blue circles, clones present at diagnosis but not relapse as red squares, clones present at relapse but not diagnosis as brown triangles; no clones were detected during remission only. Marks on the *x*-axis indicate that the respective clone was not detected in that sample. Patient IDs are provided below each plot; sample, ITD, and clinical patient details are presented in the supplementary tables 2–4. Abbreviations: after 2 cycles of chemotherapy (cy2), after end of treatment: 4th consolidation treatment (c4) or stem-cell transplantation (tx), follow-up months 1–12 (m1–12)

To study MRD in *FLT3*-ITD positive AML patients, we analyzed serial samples of 10/28 patients. A total of 18 follow-up samples was sequenced for five patients that relapsed during therapy (0.7–3.0 million, mean 1.8 million paired-end reads) and 11 follow-up samples were sequenced for five patients who remained in continuous complete remission (1.4–3.0 million, mean 2.1 million paired-end reads). Serial samples of patients in continuous complete remission were all *FLT3*-ITD negative by our assay, at all of the sampled time points (Fig. 2a, left). The relapsed patients all tested ITD positive with our assay at the time of relapse (Fig. 2a, right), whereas FA had identified only three as *FLT3*-ITD positive at relapse and two patients were considered to have lost the *FLT3*-ITD. In these two patients, our assay detected the ITDs at very low VAFs (0.06% and 0.2%, patient IDs 13 and 28, respectively; Fig. 2a), whereas no ITD could be detected in any of their preceding follow-up samples. For the other three relapsing patients, who tested *FLT3*-ITD positive at relapse also by FA, our assay detected ITDs at high VAFs at relapse (18.0–37.2%, mean 30.3%, patient IDs 21, 25, and 19; Fig. 2a) and at lower levels in at least one of the preceding follow-up samples from complete remission. Notably, in all five of the relapsed patients, there was at least one diagnostic *FLT3*-ITD clone that was detected also at relapse (Fig. 2b, blue circles). These ITD clones must have persisted during remission, at a VAF at times even below our limit of detection. Finally, *getITD* also identified, in all 10 patients, additional ITD clones that were not identified by FA: The mean number of total ITDs detected was 4.8 at diagnosis (1.3 by FA; *n* = 10) and 2.7 at relapse (0.6 by FA, *n* = 5 relapsed patients only). Those ITDs detected only by our assay were again present at very low VAFs (0.0072–2.73%, mean 0.25%) and mostly below the detection limit of conventional FA.

Importantly, sensitivity of our assay could be increased even further by applying more than the 50 ng of sample DNA that we used. However, sensitivity will increase only linearly with input DNA, and by the same factor. In accordance, closer patient monitoring might be better suited to enable earlier MRD detection, since the amount of available patient DNA is often limited, especially during complete remission. In addition to the increased sensitivity compared to conventional FA, our approach offers the benefit of identifying ITD lengths, integration sites, and sequences, all within one assay. These are not only prognostically relevant [12–15] but also allow for the delineation and unbiased monitoring of separate ITD clones within a sample. With a current read length of 250 bp and a minimum insert length of 6 bp, the maximum detectable ITD length of our assay would be 244 bp; longer ITDs, which are rare but do occur, would be missed. Read lengths are being extended continuously, though, and as *getITD* makes no assumptions on the input reads’ length, it will accommodate future changes to the preceding sequencing protocol and offer a sustainable solution to the problem of *FLT3*-ITD detection.

NGS-based assays previously described for *FLT3*-ITD MRD detection used the now discontinued Roche 454 technology [7] or Illumina protocols based on random fragmentation and subsequent enrichment or enzyme-based fragmentation and adapter tagging of target sequences [9, 10]. This subsequent enrichment is inefficient, leaving up to 25% of off-target reads in the mix and effectively increasing experiment costs (SureSelect Target Enrichment, Agilent Technologies Inc., Santa Clara, USA). Fragmentation of target sequences may break ITDs and render them undetectable [10]. To overcome these limitations, we used a two-step PCR to amplify and sequence the affected *FLT3* exons directly. We also developed a novel analysis program, *getITD*, as previously described tools were either proprietary or unable to correctly identify all of the ITDs tested. Proprietary solutions generally limit transparency as well as the benefit to and from the community. It is therefore vital that code be open source, which is why *getITD* is freely available at https://github.com/tjblaette/getitd.

Validation by serial MRD analysis revealed persisting MRD positivity only in patients who relapsed during follow-up and additional, low-allelic *FLT3*-ITD clones in all patients, at diagnosis, remission and relapse, which were undetectable by FA, highlighting our method’s potential for monitoring *FLT3*-ITD MRD in AML. In our study, our method achieved 100% sensitivity and specificity. It detected and fully annotated all of the ITDs tested and determined insertion site, length, and VAF with near perfect accuracy. Results were fully reproducible and obtained without any manual curation, making our workflow objective and easily standardized for clinical practice.

## Supplementary information

Supplementary Information
