# Supplementary material for: getITD for FLT3-ITD-based MRD monitoring in AML
Source: Leukemia. 2019 May 14;33(10):2535–9. doi: 10.1038/s41375-019-0483-z (PMC8075860; doi:10.1038/s41375-019-0483-z)
Supplement: Supplementary file 1 — Supplementary Information [file 41375_2019_483_MOESM1_ESM.pdf]

1 Subjects & Methods

1.1 Samples

For assay assessment, we sequenced AML cell lines ($n = 3$), samples from healthy volunteers ($n = 2$, from peripheral blood (PB)) and AML patient samples ($n = 57$ samples, from bone marrow aspirates (BM)). In total, and including all technical replicates, we sequenced and analyzed 90 samples.

The human AML cell lines HL-60 (*FLT3*-ITD negative control[1]), MOLM-14 (*FLT3*-ITD positive control, 21 bp ITD[1]) and PL-21 (*FLT3*-ITD positive control, 126 bp ITD[1]) were obtained from the German cell line repository DSMZ (Leibniz Institute DSMZ - German Collection of Microorganisms and Cell Cultures, Braunschweig, Germany). The 57 AML samples were derived from 28 AML patients who were all included in the AMLSG BiO Registry study (ClinicalTrials.gov identifier: NCT01252485) and gave their informed consent for both biobanking and genomic analysis of leukemia samples according to the Declaration of Helsinki.

For initial assay assessment, we sequenced 3 *FLT3*-ITD negative controls (healthy volunteers, $n = 2$; AML cell line HL-60), 2 *FLT3*-ITD positive controls (AML cell lines MOLM-14 and PL-21) and diagnosis samples of 28 AML patients who previously tested *FLT3*-ITD positive by PCR- and capillary electrophoresis-based fragment analysis (FA). To further evaluate our assay in the context of MRD monitoring of *FLT3*-ITD positive AML, we sequenced 29 follow-up samples of 10 of these AML patients, five of whom achieved continuous complete remission and five of whom relapsed from the disease. For all patients, at least one sample from complete remission, following induction therapy, and at least one additional follow-up sample, either from continued complete remission or the time of relapse,

were sequenced. All of these 10 patients were also enrolled in the AMLSG 16-10 trial (ClinicalTrials.gov identifier: NCT01477606). Within this trial, patients were treated with intensive induction chemotherapy in combination with midostaurin followed by allogeneic stem cell transplantation (HSCT) as first priority for consolidation or an age-adapted high-dose cytarabine (HDAC) based chemotherapy regimen in combination with midostaurin. A one-year maintenance therapy with midostaurin was intended for all patients, starting after chemotherapy-based consolidation or 30 days after HSCT.

A detailed sample overview is provided in Table S2, including the attained sequencing coverage and *FLT3*-ITD statistics.

1.2 FLT3-ITD detection by NGS and getITD

Next-generation sequencing: Patient samples had been enriched for mononuclear cells by Ficoll gradient centrifugation and purified cells were biobanked at −80 °C. Of these samples, genomic DNA was isolated using the AllPrep Mini Kit (Qiagen, Hilden, Germany) according to the manufacturers instructions. *FLT3* exons 14–15 were amplified by PCR, using 1 µL (50 ng) of genomic sample DNA, 20 µL of 2x KAPA HiFi HotStart ReadyMix (KAPA Biosystems, Wilmington, Massachusetts, USA) and 1.2 µL of 10 µM forward and reverse primer (forward primer: TCGTCGGCAGCGTCAGATGTGTATAAGAGACAGA + GCAATTTAGGTATGAAAGCCAGCTAC, reverse primer: CCAACGGCAGTTTTACGACTTTC + AGACAGAGAATATGTGTAGAGGCTCGGGTGCTCTG, each consisting of *FLT3* locus-specific sequence and the required sequencing adapter). The PCR comprised an initial denaturation step (95 °C 3 min), 30 amplification cycles

(denaturation 98 °C 20 s, annealing 65 °C 30 s, elongation 72 °C 1 min) and a final elongation step (72 °C 5 min). PCR products were prepared for sequencing as detailed in the Illumina 16S Metagenomics Sequencing Protocol (16S Metagenomic Sequencing Library Preparation, Illumina, San Diego, California, USA) using either single or double unique indices for sample multiplexing. The resulting libraries were sequenced to high-coverage on the Illumina MiSeq using 250 bp paired-end reads (500-cycles MiSeq Reagent Kit V2, Illumina) and 7 % phiX control spike-in (Kit V3, Illumina; coverage: 0.04–4.2 million, mean 1.6 million paired-end reads).

In total, library preparation takes 6 h, with roughly 3 h of hands-on time. Sequencing takes an additional 39 h, so that samples are fully processed within 48 h. For our validation experiments, we have aimed for a rather high coverage and pooled 8 samples per MiSeq run, generating on average around 2 million paired-end reads per sample. With this setup, assay costs amount to about 150 Euro per sample. However, more than 90 % of these costs are spent on sequencing and only a minor fraction covers the sequencing library preparation. Costs could thus be reduced to about 100 Euro per sample if 16 instead of 8 samples were pooled, which would still generate a sufficient coverage of around 1 million reads per sample.

Sample indexing: We tested our assay with both single and double unique indices for sample barcoding and multiplexing. Comparing 14 technical replicates, derived from 14/28 *FLT3*-ITD positive AML patients analyzed for assay assessment, we found significant index mis-assignment, so-called index hopping, when using single indices only[2]. Index hopping causes reads to be mis-assigned to the wrong sample in a pooled library when *FLT3*-ITD mutated reads are mis-assigned, they are then detected as false positive *contaminated* variants in these samples. For exam-

ple, in our 14 replicates sequenced with single unique indices, 22/61 *FLT3*-ITDs could be traced to index hopping. We therefore sequenced all of our AML patient samples thereafter using double unique indices, found zero ITDs attributable to index hopping, and thus recommend others also use double indexing whenever low-allelic variants are of interest. Details of the ITDs detected with either index design are provided in Table S3.

getITD: We developed the bioinformatics program *getITD* for the analysis of our assays sequencing data and the identification of respective ITDs. In brief, *getITD* aligns high-quality sequencing reads to the wild type (WT) amplicon, identifies insertions relative to this reference and then determines whether these qualify as ITDs. Insertions and ITDs are both extensively annotated and reported to the user.

By default, high-quality sequencing reads are those with an average base quality score (BQS) of at least 30, equivalent to a sequencing error probability below 0.1 %, whose sequence is not unique in a given sample. Each of these reads is aligned to the WT sequence of the target amplicon using the Needleman-Wunsch alignment algorithm[3], with alignment scores optimized for the detection of long insertions (match: 5, mismatch: -15, gap opening: -36, gap extension: -0.5). Only alignments with gap-free primer sequences and an alignment score of at least 50 % of the maximum possible score are processed further.

This alignment score, which quantifies the similarity between the reference sequence and each of the samples reads, is calculated for each read as a running sum that increases whenever bases match between the read and the reference and decreases when they do not. Very low scores are indicative of PCR artefacts and

respective reads are thus filtered out.

From passing reads, insertions relative to the reference are extracted which are (i) in-frame, (ii) at least 6 bp / 2 amino acids long and (iii) free of ambiguous bases (`N`). Inserts at the very 5' or 3' end of a read, which we call trailing inserts, are not required to be in-frame - since they are not fully covered by the read, their exact length is unknown. Detected insertions, both trailing and non-trailing, are considered ITDs if they are adjacent to a second repeat of their own sequence (Figure 1B). These repeats, the respective ITDs WT tandems, are identified by realigning the insert to the WT reference and again require an alignment score of at least 50 % of the maximum possible score. From all ITDs identified in this way, a set of high-confidence calls is obtained by filtering for those with a VAF of at least 0.006 % and two or more distinct supporting reads.

While this is the primary output of interest, all of the insertions and ITDs identified as part of the analysis are annotated and reported. Specifically, each insertion and ITD is annotated with insertion length, site, sequence and frequency. ITD frequencies are provided as VAFs and, calculated from these as shown below in equation 1, allelic ratios (ARs). Insertion sites are given as coordinates relative to the amplicon, genome, transcript and protein.

Detailed results of *getITD* are presented in Table S3 for all 210 *FLT3*-ITDs described in the manuscript.

$$AR = \frac{VAF}{100 - VAF} \qquad (1)$$

All analysis parameters are user-adjustable and can be tuned to accommodate for example single-end, merged or trimmed reads as well as reads generated from

other sequencing technologies or target regions[4]. Limits on the maximum detectable ITD length are imposed only by the sequencing data used for the analysis, not by *getITD* itself.

Code availability & details: *getITD* is implemented in python3, runs on Linux, Mac and Windows and is freely available for download at `https://github.com/tjblaette/getitd`. It may be run directly from the command-line or via a wrapper program that offers a simple graphical interface to supply input files and parameters. Analysis of a single sample with one million paired-end reads takes, depending on the computational resources available, 20 min with and up to 60 min without parallelization across multiple cores. Analysis of the data described in this manuscript was performed with default parameters and without any manual curation of results.

Fragment analysis: All patient samples were independently screened for *FLT3*-ITDs at diagnosis and relapse using the established diagnostic FA, a PCR-based amplification of *FLT3* exons 14 and 15 followed by capillary electrophoresis as described previously[5]. ITD insertion sites were determined by conventional Sanger sequencing[5]. To compare ITDs detected by FA and by NGS, which report mutation frequencies as ARs and VAFs respectively, the AR determined using the GeneScan platform was converted to the corresponding VAF as follows:

$$VAF = \frac{AR}{AR + 1} * 100 \tag{2}$$

Supplementary references

1. Quentmeier H, Reinhardt J, Zaborski M, and Drexler HG. FLT3 mutations in acute myeloid leukemia cell lines. *Leukemia* 2003; **17**: 120.

2. Kircher M, Sawyer S, and Meyer M. Double indexing overcomes inaccuracies in multiplex sequencing on the Illumina platform. *Nucleic Acids Res* 2011; **40**: e3–e3.

3. Needleman SB and Wunsch CD. A general method applicable to the search for similarities in the amino acid sequence of two proteins. *J Mol Biol* 1970; **48**: 443–453.

4. Rücker FG, Du L, Blätte TJ, Benner A, Krzykalla J, Gathmann I, et al. Prognostic Impact of Insertion Site in Acute Myeloid Leukemia (AML) with FLT3 Internal Tandem Duplication: Results from the Ratify Study (Alliance 10603). *Blood* 2018: 435 (abstract).

5. Kayser S, Schlenk RF, Londono MC, Breitenbuecher F, Wittke K, Du J, et al. Insertion of FLT3 internal tandem duplication in the tyrosine kinase domain-1 is associated with resistance to chemotherapy and inferior outcome. *Blood* 2009; **114**: 2386–2392.

MOLM-14 : HL-60<br>Expected VAF (%)	1:100<br>0.67	1:1000<br>0.067	1:10000<br>0.0067
**VAF by NGS (%)**	0.88043	0.08545	0.00483
**VAF by NGS (%)**	0.72653	0.07486	0.01509
**VAF by NGS (%)**	0.72068	0.05967	0.00073
**VAF by NGS (%)**			0.00075
**Mean**	0.77588	0.07333	0.00535
**Standard deviation**	0.09059	0.01296	0.00677
**Coefficient of variation (CV)**	0.11676	0.17677	1.26592

Table S1: Replicates of the serial dilution: For the three dilutions with expected *FLT3*-ITD frequencies 0.67 %, 0.067 % and 0.0067 %, 3–4 independent technical replicates were analyzed using our NGS-based assay. Reported here are the exact variant allele frequencies (VAFs) estimated in each replicate, also plotted in Figure 1A of the manuscript, and the standard deviation, mean and coefficient of variation (CV, standard deviation / mean) for each of the three dilutions. Note that independent serial dilutions were pipetted for all replicates.

Dilution	Patient ID	Cohort_sensitivity	Cohort_specificity	Cohort_accuracy	Cohort_reproducibility	Cohort_mrd-rl	Cohort_mrd+rl	Material	Time point	Number of ITDs (FA)	AR of ITDs (FA)	VAF of ITDs (FA / calculated)	ITD1 length & site (FA / Sanger)	ITD2 length & site (FA / Sanger)	Number of ITDs (getITD)	Total ITD VAF (getITD)	Number of FA-found ITDs (getITD)	Total VAF of FA-found ITDs (getITD)	Sample indexing	Coverage (read pairs)
-	1			yes	yes			BM	dx	1	0.192	16.10738255	54 (AS 598/599)		2	13.2897	1	12.958	double	121444
-	1				yes			BM	dx	1	0.192	16.10738255	54 (AS 598/599)		6	13.1415	1	12.68	single	502823
-	2			yes				BM	dx	1	0.617	38.15708101	72 (AS 606/607)		2	33.7071	1	33.695	double	1139145
-	3			yes	yes			BM	dx	1	0.789	44.10285075	21 (AS 600/601)		1	43.489	1	43.489	double	1143537
-	3				yes			BM	dx	1	0.789	44.10285075	21 (AS 600/601)		3	44.4894	1	44.45	single	283201
-	4			yes	yes			BM	dx	1	17.169	94.49611976	39 (AS 593)		1	94.653	1	94.653	double	143104
-	4				yes			BM	dx	1	17.169	94.49611976	39 (AS 593)		6	89.1975	1	89.111	single	642035
-	5			yes	yes			BM	dx	1	0.861	46.26544868	42 (AS 602)		1	44.728	1	44.728	double	2092251
-	5				yes			BM	dx	1	0.861	46.26544868	42 (AS 602)		3	47.0621	1	47.007	single	321840
-	6			yes	yes	yes		BM	dx	1	0.552	35.56701031	27 (AS 599)		5	37.2114	1	34.127	double	2110204
-	6				yes			BM	dx	1	0.552	35.56701031	27 (AS 599)		5	38.6549	1	33.755	single	271565
-	6					yes		BM	cr_cy2_c1						0	0	0	0	double	2668487
-	6					yes		BM	cr_fu_m11						0	0	0	0	double	1987005
-	7			yes				BM	dx	1	0.752	42.92237443	33 (AS 596/597)		5	43.7485	1	43.702	double	1602251
-	8			yes	yes	yes		BM	dx	1	0.686	40.68801898	36 (AS 608/609)		2	44.7312	1	44.718	double	1358468
-	8				yes			BM	dx	1	0.686	40.68801898	36 (AS 608/609)		1	46.784	1	46.784	single	285460
-	8					yes		BM	cr_cy2_c1						0	0	0	0	double	2415099
-	8					yes		BM	cr_fu_m3						0	0	0	0	double	2950217
-	8					yes		BM	cr_fu_m12						0	0	0	0	double	1895959
-	9			yes				BM	dx	1	0.103	9.338168631	48 (AS 610)		1	3.4012	1	3.4012	double	779831
-	10			yes				BM	dx	2	0.347	25.76095026	24 (AS 598)	33 (AS 594)	12	25.5057	2	24.677	double	2324362
-	11			yes	yes			BM	dx	1	0.452	31.12947658	45 (AS 590/591)		1	24.702	1	24.702	double	182304
-	11				yes			BM	dx	1	0.452	31.12947658	45 (AS 590/591)		3	27.636	1	27.6	single	1344506
-	12			yes	yes	yes		BM	dx	1	0.768	43.43891403	45 (AS 598)		2	42.2052	1	41.874	double	2076011
-	12				yes			BM	dx	1	0.768	43.43891403	45 (AS 598)		2	42.3822	1	42.105	single	543655
-	12					yes		BM	cr_cy2_c1						0	0	0	0	double	1856671
-	12					yes		BM	cr_fu_m12						0	0	0	0	double	1887112
-	13			yes	yes		yes	BM	dx	2	0.276	21.63009404	60 (AS 613)	39 (AS 595/596)	2	22.2246	2	22.2246	double	1423217
-	13				yes			BM	dx	2	0.276	21.63009404	60 (AS 613)	39 (AS 595/596)	3	22.2663	2	22.2529	single	383536
-	13						yes	BM	cr_cy2_c1						0	0	0	0	double	1708522
-	13						yes	BM	cr_eot_c4						0	0	0	0	double	2962904
-	13						yes	BM	cr_fu_m3						0	0	0	0	double	2013235
-	13						yes	BM	cr_fu_m6						0	0	0	0	double	2220894
-	13						yes	BM	cr_fu_m9						0	0	0	0	double	1426831
-	13						yes	BM	rl_fu_m11	0	0	0			2	0.21863	0	0	double	1888212
-	14			yes				BM	dx	2	0.765	43.3427762	18 (AS 603)	30 (AS 593)	2	42.581	2	42.581	double	2474806
-	15			yes	yes			BM	dx	1	0.525	34.42622951	24 (AS 601/602)		1	31.827	1	31.827	double	38989
-	15				yes			BM	dx	1	0.525	34.42622951	24 (AS 601/602)		9	31.0303	1	30.846	single	549621
-	16			yes				BM	dx	1	0.863	46.32313473	18 (AS 598)		5	42.8743	1	41.651	double	2601573
-	17			yes				BM	dx	2	0.412	29.17847025	54 (AS 613)	90 (AS 606)	11	29.2862	2	28.646	double	1203484
-	18			yes	yes	yes		BM	dx	1	5.419	84.42124942	27 (AS 608)		2	78.7967	1	78.789	double	1160877
-	18				yes			BM	dx	1	5.419	84.42124942	27 (AS 608)		1	77.659	1	77.659	single	237902
-	18					yes		BM	cr_cy2_c1						0	0	0	0	double	2331331
-	18					yes		BM	cr_eot_c4						0	0	0	0	double	2178063
-	19			yes			yes	BM	dx	2	0.195	16.31799163	36 (AS 596)	93 (AS 611/612)	16	13.6331	2	8.0268	double	1268768
-	19						yes	BM	cr_cy2_i2						4	0.654645	0	0	double	1480411
-	19						yes	BM	cr_eot_tx						1	0.03156	0	0	double	1241329
-	19						yes	BM	rl_fu_m4	1	0.299	23.01770593	93 (AS 611/612)		8	17.9518	1	17.484	double	1071694
-	20			yes				BM	dx	1	0.51	33.77483444	39 (AS 593)		3	28.6368	1	28.364	double	1454110
-	21			yes	yes		yes	BM	dx	1	0.632	38.7254902	87 (AS 609/610)		4	38.2656	1	38.089	double	2486855
-	21				yes			BM	dx	1	0.632	38.7254902	87 (AS 609/610)		3	34.866	1	34.724	single	238719
-	21						yes	BM	cr_cy2_i2						1	0.93816	0	0	double	2709486
-	21						yes	BM	cr_eot_tx						0	0	0	0	double	2857166
-	21						yes	BM	cr_fu_m3						1	0.060832	0	0	double	1131351
-	21						yes	BM	rl_fu_m5	1	0.646	39.24665857	87 (AS 609/610)		1	35.688	1	35.688	double	745374
-	22			yes				BM	dx	1	0.892	47.14587738	39 (AS 611/612)		3	46.1544	1	46.136	double	1095411
-	23			yes		yes		BM	dx	1	0.76	43.18181818	54 (AS 600)		2	41.2423	1	41.156	double	1633097
-	23					yes		BM	cr_cy2_c1						0	0	0	0	double	1942717
-	23					yes		BM	cr_eot_c4						0	0	0	0	double	1394662
-	24			yes				BM	dx	1	0.788	44.07158837	66 (AS 610)		1	39.744	1	39.744	double	2526191
-	25			yes	yes		yes	BM	dx	2	0.398	28.46924177	42 (AS 590)	60 (AS 598)	7	29.083	2	27.903	double	736243
-	25				yes			BM	dx	2	0.398	28.46924177	42 (AS 590)	60 (AS 598)	8	29.5654	2	28.573	single	789474
-	25						yes	BM	cr_cy2_c1						6	2.22909	0	0	double	2705254
-	25						yes	BM	cr_eot_c4						1	0.63748	0	0	double	1862886
-	25						yes	BM	rl_fu_m1	1	0.744	42.66055046	60 (AS 598)		2	37.1815	1	37.103	double	1478562
-	26			yes				BM	dx	1	0.178	15.11035654	36 (AS 608/609)		3	15.5536	1	15.505	double	2254869
-	27			yes				BM	dx	1	0.861	46.26544868	96 (AS 613)		2	48.3012	1	48.29	double	1160877
-	28			yes	yes		yes	BM	dx	1	0.386	27.84992785	198 (AS 614/615)		6	10.326	1	9.9464	double	1022779
-	28				yes			BM	dx	1	0.386	27.84992785	198 (AS 614/615)		8	20.9549	1	20.593	single	340073
-	28						yes	BM	cr_cy2_c1						0	0	0	0	double	1483295
-	28						yes	BM	rl_fu_m4	0	0	0			1	0.055593	0	0	double	2069770
-	HL-60		yes					cell_line	-	KNOWN 0	KNOWN 0	0			0	0	0	0	double	4164257
-	healthy_volunteer1		yes					PB	-						0	0	0	0	double	1137581
-	healthy_volunteer2		yes					PB	-						0	0	0	0	double	2441834
10-0	MOLM-14	yes						cell_line	-	1	KNOWN 2, FA 2.451	KNOWN 66.7, FA 71.02289192	21 (AS 599)		1	64.979	1	64.979	double	1797368
10-1	MOLM-14	yes						cell_line	-	1	KNOWN 0.0718	KNOWN 6.7	21 (AS 599)		1	8.6377	1	8.6377	double	2698207
10-2	MOLM-14	yes						cell_line	-	1	KNOWN 0.00675	KNOWN 0.67	21 (AS 599)		1	0.72068	1	0.72068	double	1686834
10-2	MOLM-14	yes						cell_line	-	1	KNOWN 0.00675	KNOWN 0.67	21 (AS 599)		1	0.72653	1	0.72653	double	1629789
10-2	MOLM-14	yes						cell_line	-	1	KNOWN 0.00675	KNOWN 0.67	21 (AS 599)		1	0.88043	1	0.88043	double	2681959
10-3	MOLM-14	yes						cell_line	-	1	KNOWN 0.000670	KNOWN 0.067	21 (AS 599)		1	0.059666	1	0.059666	double	1139435
10-3	MOLM-14	yes						cell_line	-	1	KNOWN 0.000670	KNOWN 0.067	21 (AS 599)		1	0.074857	1	0.074857	double	1261903
10-3	MOLM-14	yes						cell_line	-	1	KNOWN 0.000670	KNOWN 0.067	21 (AS 599)		1	0.085454	1	0.085454	double	2850841
10-4	MOLM-14	yes						cell_line	-	1	KNOWN 0.0000670	KNOWN 0.0067	21 (AS 599)		1	0.0048347	1	0.0048347	double	2408074
10-4	MOLM-14	yes						cell_line	-	1	KNOWN 0.0000670	KNOWN 0.0067	21 (AS 599)		1	0.015087	1	0.015087	double	1609750
10-4	MOLM-14	yes						cell_line	-	1	KNOWN 0.0000670	KNOWN 0.0067	21 (AS 599)		1	0.00073096	1	0.00073096	double	1742353
10-4	MOLM-14	yes						cell_line	-	1	KNOWN 0.0000670	KNOWN 0.0067	21 (AS 599)		1	0.00074673	1	0.00074673	double	1562426
10-0	PL-21	yes						cell_line	-	1	KNOWN 0.5	KNOWN 33.3	KNOWN 126 (AS 613)		1	18.641	1	18.641	double	2249360
10-1	PL-21	yes						cell_line	-	1	KNOWN 0.0341	KNOWN 3.3	KNOWN 126 (AS 613)		1	2.6228	1	2.6228	double	2353158
10-2	PL-21	yes						cell_line	-	1	KNOWN 0.00331	KNOWN 0.33	KNOWN 126 (AS 613)		1	0.26	1	0.26	double	2198641
10-3	PL-21	yes						cell_line	-	1	KNOWN 0.000330	KNOWN 0.033	KNOWN 126 (AS 613)		1	0.014983	1	0.014983	double	1432778

Table S2: Sample overview: Listed are all of the samples processed and described in the manuscript with results obtained by fragment analysis (FA) and our NGS-based assay and *getITD*-based analysis.

Dilution	Patient ID	Time point	Sample indexing	Index hopping artefact	ITD length (bp)	Start (bp, in amplicon)	VAF	AR	Coverage (read pairs)	ITD supporting (read pairs)	Trailing	Insert sequence	Insert domains	Start (bp, in chr13)	Start (bp, in transcript)	Start (AA, in protein)	End (bp, in chr13)	End (bp, in transcript)	End (AA, in protein)	Insertion site (bp, in chr13)	Insertion site (bp, in transcript)	Insertion site (AA, in protein)	Insertion site domain
-	1	dx	double	no	21	79	0.332	0.00333	74462	247	False	[illegible]	[illegible]	28608274	1782	594	28608254	1802	601	28608253	1803	601	exon14_JMD_zipperMotif
-	1	dx	double	no	54	38	12.958	0.14887	74462	9649	False	[illegible]	[illegible]	28608315	1741	581	28608262	1794	598	28608261	1795	599	exon14_JMD_zipperMotif
-	1	dx	single	no	21	79	0.386	0.00387	461135	1778	False	[illegible]	[illegible]	28608274	1782	594	28608254	1802	601	28608253	1803	601	exon14_JMD_zipperMotif
-	1	dx	single	yes	39	56	0.028	0.00028	436196	124	False	[illegible]	[illegible]	28608297	1759	587	28608259	1797	599	28608258	1798	600	exon14_JMD_zipperMotif
-	1	dx	single	yes	45	90	0.007	0.00007	436196	32	False	[illegible]	[illegible]	28608263	1793	598	28608219	1837	613	28608218	-	-	intron14_spliceDonor
-	1	dx	single	no	54	38	12.680	0.14521	436196	55308	False	[illegible]	[illegible]	28608315	1741	581	28608262	1794	598	28608261	1795	599	exon14_JMD_zipperMotif
-	1	dx	single	yes	54	70	0.022	0.00022	436196	97	False	[illegible]	[illegible]	28608283	1773	591	28608230	1826	609	28608229	1827	609	exon14_JMD_hingeRegion
-	1	dx	single	yes	66	73	0.018	0.00018	436196	78	False	[illegible]	[illegible]	28608280	1776	592	28608215	-	-	28608214	-	-	intron14
-	2	dx	double	no	36	86	0.012	0.00012	1004613	122	False	[illegible]	[illegible]	28608267	1789	597	28608232	1824	608	28608231	1825	609	exon14_JMD_hingeRegion
-	2	dx	double	no	72	44	33.695	0.50818	1004613	338502	False	[illegible]	[illegible]	28608309	1747	583	28608238	1818	606	28608237	1819	607	exon14_JMD_hingeRegion
-	3	dx	double	no	21	77	43.489	0.76957	1054593	458637	False	[illegible]	[illegible]	28608276	1780	594	28608256	1800	600	28608255	1801	601	exon14_JMD_zipperMotif
-	3	dx	single	no	21	77	44.450	0.80018	114312	50812	False	[illegible]	[illegible]	28608276	1780	594	28608256	1800	600	28608255	1801	601	exon14_JMD_zipperMotif
-	3	dx	single	yes	42	61	0.023	0.00023	114312	26	False	[illegible]	[illegible]	28608292	1764	588	28608251	1805	602	28608250	1806	602	exon14_JMD_zipperMotif
-	3	dx	single	yes	54	25	0.017	0.00017	114312	19	False	[illegible]	[illegible]	28608328	1728	576	28608275	1781	594	28608274	1782	594	exon14_JMD_zipperMotif
-	4	dx	double	no	39	37	94.653	17.70200	65705	62192	False	[illegible]	[illegible]	28608316	1740	580	28608278	1778	593	28608277	1779	593	exon14_JMD_zipperMotif
-	4	dx	single	yes	179	46	0.019	0.00019	51962	10	True	[illegible]	[illegible]	28608307	1749	583	28608129	-	-	28608128	1838	613	exon15_TKD1_beta1Sheet
-	4	dx	single	no	39	37	89.111	8.18360	482100	429605	False	[illegible]	[illegible]	28608316	1740	580	28608278	1778	593	28608277	1779	593	exon14_JMD_zipperMotif
-	4	dx	single	yes	45	90	0.007	0.00007	482100	32	False	[illegible]	[illegible]	28608263	1793	598	28608219	1837	613	28608218	-	-	intron14_spliceDonor
-	4	dx	single	yes	54	41	0.026	0.00026	482100	127	False	[illegible]	[illegible]	28608312	1744	582	28608259	1797	599	28608258	1798	600	exon14_JMD_zipperMotif
-	4	dx	single	yes	54	70	0.020	0.00020	482100	95	False	[illegible]	[illegible]	28608283	1773	591	28608230	1826	609	28608229	1827	609	exon14_JMD_hingeRegion
-	4	dx	single	yes	66	73	0.015	0.00015	482100	70	False	[illegible]	[illegible]	28608280	1776	592	28608215	-	-	28608214	-	-	intron14
-	5	dx	double	no	42	61	44.728	0.80923	1849947	827446	False	[illegible]	[illegible]	28608292	1764	588	28608251	1805	602	28608250	1806	602	exon14_JMD_zipperMotif
-	5	dx	single	yes	21	77	0.044	0.00044	139798	61	False	[illegible]	[illegible]	28608276	1780	594	28608256	1800	600	28608255	1801	601	exon14_JMD_zipperMotif
-	5	dx	single	no	42	61	47.007	0.88704	139798	65715	False	[illegible]	[illegible]	28608292	1764	588	28608251	1805	602	28608250	1806	602	exon14_JMD_zipperMotif
-	5	dx	single	yes	54	25	0.011	0.00011	139798	16	False	[illegible]	[illegible]	28608328	1728	576	28608275	1781	594	28608274	1782	594	exon14_JMD_zipperMotif
-	6	dx	double	no	21	65	0.008	0.00008	1837396	147	False	[illegible]	[illegible]	28608288	1768	590	28608268	1788	596	28608267	1789	597	exon14_JMD_zipperMotif
-	6	dx	double	no	27	66	34.127	0.51807	1837396	627048	False	[illegible]	[illegible]	28608287	1769	590	28608261	1795	599	28608260	1796	599	exon14_JMD_zipperMotif
-	6	dx	double	no	30	61	0.058	0.00058	1837396	1071	False	[illegible]	[illegible]	28608292	1764	588	28608263	1793	598	28608262	1794	598	exon14_JMD_zipperMotif
-	6	dx	double	no	33	29	0.285	0.00286	1837396	5241	False	[illegible]	[illegible]	28608324	1732	578	28608292	1764	588	28608291	1765	589	exon14_JMD_switchMotif
-	6	dx	double	no	66	42	2.733	0.02810	1837396	50215	False	[illegible]	[illegible]	28608311	1745	582	28608246	1810	604	28608245	1811	604	exon14_JMD_hingeRegion
-	6	dx	single	no	21	71	0.012	0.00012	120472	14	False	[illegible]	[illegible]	28608282	1774	592	28608262	1794	598	28608261	1795	599	exon14_JMD_zipperMotif
-	6	dx	single	no	27	66	33.755	0.50955	120472	40665	False	[illegible]	[illegible]	28608287	1769	590	28608261	1795	599	28608260	1796	599	exon14_JMD_zipperMotif
-	6	dx	single	no	30	61	0.061	0.00061	120472	74	False	[illegible]	[illegible]	28608292	1764	588	28608263	1793	598	28608262	1794	598	exon14_JMD_zipperMotif
-	6	dx	single	no	33	29	0.328	0.00329	120472	395	False	[illegible]	[illegible]	28608324	1732	578	28608292	1764	588	28608291	1765	589	exon14_JMD_switchMotif
-	6	dx	single	no	66	42	4.499	0.04711	120472	5420	False	[illegible]	[illegible]	28608311	1745	582	28608246	1810	604	28608245	1811	604	exon14_JMD_hingeRegion
-	7	dx	double	no	18	67	0.024	0.00024	1428432	345	False	[illegible]	[illegible]	28608286	1770	590	28608269	1787	596	28608268	1788	596	exon14_JMD_zipperMotif
-	7	dx	double	no	27	67	0.007	0.00007	1428432	98	False	[illegible]	[illegible]	28608286	1770	590	28608260	1796	599	28608259	1797	599	exon14_JMD_zipperMotif
-	7	dx	double	no	33	53	43.702	0.77626	1428432	624249	False	[illegible]	[illegible]	28608300	1756	586	28608268	1788	596	28608267	1789	597	exon14_JMD_zipperMotif
-	7	dx	double	no	48	89	0.006	0.00006	1428432	89	False	[illegible]	[illegible]	28608264	1792	598	28608217	-	-	28608216	-	-	intron14
-	7	dx	double	no	66	20	0.009	0.00009	1428432	132	False	[illegible]	[illegible]	28608333	1723	575	28608268	1788	596	28608267	1789	597	exon14_JMD_zipperMotif
-	8	dx	double	no	36	86	44.718	0.80891	1198394	535898	False	[illegible]	[illegible]	28608267	1789	597	28608232	1824	608	28608231	1825	609	exon14_JMD_hingeRegion
-	8	dx	double	no	72	50	0.013	0.00013	1198394	158	False	[illegible]	[illegible]	28608303	1753	585	28608232	1824	608	28608231	1825	609	exon14_JMD_hingeRegion
-	8	dx	single	no	36	86	46.784	0.87913	133939	62662	False	[illegible]	[illegible]	28608267	1789	597	28608232	1824	608	28608231	1825	609	exon14_JMD_hingeRegion
-	9	dx	double	no	48	79	3.401	0.03521	746478	25389	False	[illegible]	[illegible]	28608274	1782	594	28608227	1829	610	28608226	1830	610	exon14_TKD1_beta1Sheet
-	10	dx	double	no	15	76	0.013	0.00013	2023998	264	False	[illegible]	[illegible]	28608277	1779	593	28608263	1793	598	28608262	1794	598	exon14_JMD_zipperMotif
-	10	dx	double	no	21	72	0.026	0.00026	2023998	520	False	[illegible]	[illegible]	28608281	1775	592	28608261	1795	599	28608260	1796	599	exon14_JMD_zipperMotif
-	10	dx	double	no	24	67	8.717	0.09550	2023998	176438	False	[illegible]	[illegible]	28608286	1770	590	28608263	1793	598	28608262	1794	598	exon14_JMD_zipperMotif
-	10	dx	double	no	27	38	0.009	0.00009	2023998	175	False	[illegible]	[illegible]	28608315	1741	581	28608289	1767	589	28608288	1768	590	exon14_JMD_switchMotif
-	10	dx	double	no	27	59	0.420	0.00422	2023998	8500	False	[illegible]	[illegible]	28608294	1762	588	28608268	1788	596	28608267	1789	597	exon14_JMD_zipperMotif
-	10	dx	double	no	30	61	0.006	0.00006	2023998	129	False	[illegible]	[illegible]	28608292	1764	588	28608263	1793	598	28608262	1794	598	exon14_JMD_zipperMotif
-	10	dx	double	no	33	45	15.960	0.18991	2023998	323033	False	[illegible]	[illegible]	28608308	1748	583	28608276	1780	594	28608275	1781	594	exon14_JMD_zipperMotif
-	10	dx	double	no	42	93	0.012	0.00012	2023998	238	False	[illegible]	[illegible]	28608260	1796	599	28608219	1837	613	28608218	-	-	intron14_spliceDonor
-	10	dx	double	no	69	63	0.016	0.00016	2023998	333	False	[illegible]	[illegible]	28608290	1766	589	28608222	1834	612	28608221	1835	612	exon14_TKD1_beta1Sheet
-	10	dx	double	no	81	42	0.155	0.00156	2023998	3145	False	[illegible]	[illegible]	28608311	1745	582	28608231	1825	609	28608230	1826	609	exon14_JMD_hingeRegion
-	10	dx	double	no	90	39	0.085	0.00085	2023998	1723	False	[illegible]	[illegible]	28608314	1742	581	28608225	1831	611	28608224	1832	611	exon14_TKD1_beta1Sheet
-	10	dx	double	no	96	39	0.086	0.00086	2023998	1739	False	[illegible]	[illegible]	28608314	1742	581	28608219	1837	613	28608218	-	-	intron14_spliceDonor
-	11	dx	double	no	45	23	24.702	0.32806	82612	20407	False	[illegible]	[illegible]	28608330	1726	576	28608286	1770	590	28608285	1771	591	exon14_JMD_switchMotif
-	11	dx	single	yes	21	81	0.019	0.00019	1229504	228	False	[illegible]	[illegible]	28608272	1784	595	28608252	1804	602	28608251	1805	602	exon14_JMD_zipperMotif
-	11	dx	single	no	45	23	27.600	0.38122	1165064	321560	False	[illegible]	[illegible]	28608330	1726	576	28608286	1770	590	28608285	1771	591	exon14_JMD_switchMotif
-	11	dx	single	yes	48	72	0.017	0.00017	1165058	203	False	[illegible]	[illegible]	28608281	1775	592	28608234	1822	608	28608233	1823	608	exon14_JMD_hingeRegion
-	12	dx	double	no	24	67	0.331	0.00332	1825658	6047	False	[illegible]	[illegible]	28608286	1770	590	28608263	1793	598	28608262	1794	598	exon14_JMD_zipperMotif
-	12	dx	double	no	45	46	41.874	0.72040	1825658	764482	False	[illegible]	[illegible]	28608307	1749	583	28608263	1793	598	28608262	1794	598	exon14_JMD_zipperMotif
-	12	dx	single	no	24	67	0.277	0.00278	244605	678	False	[illegible]	[illegible]	28608286	1770	590	28608263	1793	598	28608262	1794	598	exon14_JMD_zipperMotif
-	12	dx	single	no	45	46	42.105	0.72726	244605	102992	False	[illegible]	[illegible]	28608307	1749	583	28608263	1793	598	28608262	1794	598	exon14_JMD_zipperMotif
-	13	dx	double	no	39	45	1.910	0.01947	1320222	25211	False	[illegible]	[illegible]	28608308	1748	583	28608270	1786	596	28608269	1787	596	exon14_JMD_zipperMotif
-	13	dx	double	no	60	77	20.315	0.25494	1320222	268204	False	[illegible]	[illegible]	28608276	1780	594	28608217	-	-	28608216	-	-	intron14
-	13	rl_m11	double	no	21	72	0.016	0.00016	1787033	282	False	[illegible]	[illegible]	28608281	1775	592	28608261	1795	599	28608260	1796	599	exon14_JMD_zipperMotif
-	13	rl_m11	double	no	60	77	0.203	0.00203	1787033	3625	False	[illegible]	[illegible]	28608276	1780	594	28608217	-	-	28608216	-	-	intron14
-	13	dx	single	yes	197	32	0.013	0.00013	156759	21	True	[illegible]	[illegible]	28608321	1735	579	28608125	1841	614	28608124	1842	614	exon15_TKD1_beta1Sheet
-	13	dx	single	no	39	45	1.830	0.01864	201651	3690	False	[illegible]	[illegible]	28608308	1748	583	28608270	1786	596	28608269	1787	596	exon14_JMD_zipperMotif
-	13	dx	single	no	60	77	20.423	0.25664	201651	41184	False	[illegible]	[illegible]	28608276	1780	594	28608217	-	-	28608216	-	-	intron14
-	14	dx	double	no	18	87	15.753	0.18699	2117395	333546	False	[illegible]	[illegible]	28608266	1790	597	28608249	1807	603	28608248	1808	603	exon14_JMD_zipperMotif
-	14	dx	double	no	30	46	26.828	0.36664	2117395	568064	False	[illegible]	[illegible]	28608307	1749	583	28608278	1778	593	28608277	1779	593	exon14_JMD_zipperMotif
-	15	dx	double	no	24	77	31.827	0.46686	25997	8274	False	[illegible]	[illegible]	28608276	1780	594	28608253	1803	601	28608252	1804	602	exon14_JMD_zipperMotif
-	15	dx	single	no	18	70	0.064	0.00064	467648	298	False	[illegible]	[illegible]	28608283	1773	591	28608266	1790	597	28608265	1791	597	exon14_JMD_zipperMotif
-	15	dx	single	yes	21	81	0.022	0.00022	467648	102	False	[illegible]	[illegible]	28608272	1784	595	28608252	1804	602	28608251	1805	602	exon14_JMD_zipperMotif
-	15	dx	single	no	24	77	30.846	0.44605	467648	144252	False	[illegible]	[illegible]	28608276	1780	594	28608253	1803	601	28608252	1804	602	exon14_JMD_zipperMotif
-	15	dx	single	yes	39	37	0.007	0.00007	467648	33	False	[illegible]	[illegible]	28608316	1740	580	28608278	1778	593	28608277	1779	593	exon14_JMD_zipperMotif
-	15	dx	single	yes	45	23	0.018	0.00018	467648	84	False	[illegible]	[illegible]	28608330	1726	576	28608286	1770	590	28608285	1771	591	exon14_JMD_switchMotif
-	15	dx	single	yes	48	72	0.033	0.00033	467648	154	False	[illegible]	[illegible]	28608281	1775	592	28608234	1822	608	28608233	1823	608	exon14_JMD_hingeRegion
-	15	dx	single	yes	57	78	0.010	0.00010	467648	48	False	[illegible]	[illegible]	28608275	1781	594	28608219	1837	613	28608218	-	-	intron14_spliceDonor
-	15	dx	single	no	63	37	0.022	0.00022	467648	103	False	[illegible]	[illegible]	28608316	1740	580	28608254	1802	601	28608253	1803	601	exon14_JMD_zipperMotif
-	15	dx	single	yes	99	36	0.009	0.00009	467648	40	False	[illegible]	[illegible]	28608317	1739	580	28608219	1837	613	28608218	-	-	intron14_spliceDonor
-	16	dx	double	no	18	73	41.651	0.71383	2223563	926144	False	[illegible]	[illegible]	28608280	1776	592	28608263	1793	598	28608262	1794	598	exon14_JMD_zipperMotif
-	16	dx	double	no	33	53	0.015	0.00015	2223563	343	False	[illegible]	[illegible]	28608300	1756	586	28608268	1788	596	28608267	1789	597	exon14_JMD_zipperMotif
-	16	dx	double	no	48	43	0.012	0.00012	2223563	260	False	[illegible]	[illegible]	28608310	1746	582	28608263	1793	598	28608262	1794	598	exon14_JMD_zipperMotif
-	16	dx	double	no	78	23	1.187	0.01201	2223563	26387	False	[illegible]	[illegible]	28608330	1726	576	28608253	1803	601	28608252	1804	602	exon14_JMD_zipperMotif
-	16	dx	double	no	96	13	0.009	0.00009	2223563	210	False	[illegible]	[illegible]	28608340	1716	572	28608245	1811	604	28608244	1812	604	exon14_JMD_hingeRegion
-	17	dx	double	no	33	52	0.040	0.00040	1100567	441	False	[illegible]	[illegible]	28608301	1755	585	28608269	1787	596	28608268	1788	596	exon14_JMD_zipperMotif
-	17	dx	double	no	36	44	0.031	0.00031	1100567	344	False	[illegible]	[illegible]	28608309	1747	583	28608274	1782	594	28608273	1783	595	exon14_JMD_zipperMotif
-	17	dx	double	no	42	39	0.038	0.00038	1100567	418	False	[illegible]	[illegible]	28608314	1742	581	28608273	1783	595	28608272	1784	595	exon14_JMD_zipperMotif
-	17	dx	double	no	42	86	0.016	0.00016	1100567	176	False	[illegible]	[illegible]	28608267	1789	597	28608226	1830	610	28608225	1831	611	exon14_TKD1_beta1Sheet
-	17	dx	double	no	51	25	0.013	0.00013	1100567	140	False	[illegible]	[illegible]	28608328	1728	576	28608278	1778	593	28608277	1779	593	exon14_JMD_zipperMotif
-	17	dx	double	no	54	82	16.566	0.19855	1100567	182316	False	[illegible]	[illegible]	28608271	1785	595	28608218	-	-	28608217	-	-	intron14_spliceDonor
-	17	dx	double	no	66	34	0.007	0.00007	1100567	73	False	[illegible]	[illegible]	28608319	1737	579	28608254	1802	601	28608253	1803	601	exon14_JMD_zipperMotif
-	17	dx	double	no	69	11	0.229	0.00229	1100567	2515	False	[illegible]	[illegible]	28608342	1714	572	28608274	1782	594	28608273	1783	595	exon14_JMD_zipperMotif
-	17	dx	double	no	69	70	0.011	0.00011	1100567	121	False	[illegible]	[illegible]	28608283	1773	591	28608215	-	-	28608214	-	-	intron14
-	17	dx	double	no	78	37	0.256	0.00257	1100567	2818	False	[illegible]	[illegible]	28608316	1740	580	28608239	1817	606	28608238	1818	606	exon14_JMD_hingeRegion
-	17	dx	double	no	90	25	12.080	0.13740	1100567	132944	False	[illegible]	[illegible]	28608328	1728	576	28608239	1817	606	28608238	1818	606	exon14_JMD_hingeRegion
-	18	dx	double	no	18	71	0.008	0.00008	1035759	80	False	[illegible]	[illegible]	28608282	1774	592	28608265	1791	597	28608264	1792	598	exon14_JMD_zipperMotif
-	18	dx	double	no	27	94	78.789	3.71450	1072084	844685	False	[illegible]	[illegible]	28608259	1797	599	28608233	1823	608	28608232	1824	608	exon14_JMD_hingeRegion
-	18	dx	single	no	27	94	77.659	3.47610	101431	78770	False	[illegible]	[illegible]	28608259	1797	599	28608233	1823	608	28608232	1824	608	exon14_JMD_hingeRegion
-	19	cr-cy2-i2	double	no	36	49	0.475	0.00477	1444299	6861	False	[illegible]	[illegible]	28608304	1752	584	28608269	1787	596	28608268	1788	596	exon14_JMD_zipperMotif
-	19	cr-cy2-i2	double	no	45	34	0.011	0.00011	1444299	164	False	[illegible]	[illegible]	28608319	1737	579	28608275	1781	594	28608274	1782	594	exon14_JMD_zipperMotif
-	19	cr-cy2-i2	double	no	63	36	0.020	0.00020	1444299	288	False	[illegible]	[illegible]	28608317	1739	580	28608255	1801	601	28608254	1802	601	exon14_JMD_zipperMotif
-	19	cr-cy2-i2	double	no	93	38	0.148	0.00149	1444299	2142	False	[illegible]	[illegible]	28608315	1741	581	28608223	1833	611	28608222	1834	612	exon14_TKD1_beta1Sheet
-	19	cr-eot-tx	double	no	93	38	0.032	0.00032	1213551	383	False	[illegible]	[illegible]	28608315	1741	581	28608223	1833	611	28608222	1834	612	exon14_TKD1_beta1Sheet
-	19	dx	double	no	194	35	0.103	0.00103	981639	1007	True	[illegible]	[illegible]	28608318	1738	580	28608125	1841	614	28608124	1842	614	exon15_TKD1_beta1Sheet
-	19	dx	double	no	21	77	0.015	0.00015	1136406	173	False	[illegible]	[illegible]	28608276	1780	594	28608256	1800	600	28608255	1801	601	exon14_JMD_zipperMotif
-	19	dx	double	no	30	61	0.012	0.00012	1136406	135	False	[illegible]	[illegible]	28608292	1764	588	28608263	1793	598	28608262	1794	598	exon14_JMD_zipperMotif
-	19	dx	double	no	36	49	4.520	0.04734	1136406	51361	False	[illegible]	[illegible]	28608304	1752	584	28608269	1787	596	28608268	1788	596	exon14_JMD_zipperMotif
-	19	dx	double	no	45	34	1.841	0.01876	1136406	20921	False	[illegible]	[illegible]	28608319	1737	579	28608275	1781	594	28608274	1782	594	exon14_JMD_zipperMotif
-	19	dx	double	no	51	25	0.007	0.00007	1136406	85	False	[illegible]	[illegible]	28608328	1728	576	28608278	1778	593	28608277	1779	593	exon14_JMD_zipperMotif
-	19	dx	double	no	54	37	0.255	0.00256	1136406	2898	False	[illegible]	[illegible]	28608316	1740	580	28608263	1793	598	28608262	1794	598	exon14_JMD_zipperMotif
-	19	dx	double	no	54	81	0.017	0.00017	1136406	197	False	[illegible]	[illegible]	28608272	1784	595	28608219	1837	613	28608218	-	-	intron14_spliceDonor
-	19	dx	double	no	57	28	0.279	0.00280	1136406	3168	False	[illegible]	[illegible]	28608325	1731	577	28608269	1787	596	28608268	1788	596	exon14_JMD_zipperMotif
-	19	dx	double	no	57	78	0.016	0.00016	1136406	179	False	[illegible]	[illegible]	28608275	1781	594	28608219	1837	613	28608218	-	-	intron14_spliceDonor
-	19	dx	double	no	60	75	0.218	0.00218	1136406	2473	False	[illegible]	[illegible]	28608278	1778	593	28608219	1837	613	28608218	-	-	intron14_spliceDonor
-	19	dx	double	no	63	36	2.728	0.02804	1136406	31000	False	[illegible]	[illegible]	28608317	1739	580	28608255	1801	601	28608254	1802	601	exon14_JMD_zipperMotif
-	19	dx	double	no	72	45	0.050	0.00050	1136406	570	False	[illegible]	[illegible]	28608308	1748	583	28608237	1819	607	28608236	1820	607	exon14_JMD_hingeRegion
-	19	dx	double	no	87	48	0.057	0.00057	1136406	649	False	[illegible]	[illegible]	28608305	1751	584	28608219	1837	613	28608218	-	-	intron14_spliceDonor
-	19	dx	double	no	93	38	3.507	0.03635	1136406	39856	False	[illegible]	[illegible]	28608315	1741	581	28608223	1833	611	28608222	1834	612	exon14_TKD1_beta1Sheet
-	19	dx	double	no	96	31	0.008	0.00008	1136406	96	False	[illegible]	[illegible]	28608322	1734	578	28608227	1829	610	28608226	1830	610	exon14_TKD1_beta1Sheet
-	19	rl_m4	double	no	194	35	0.021	0.00021	772371	163	True	[illegible]	[illegible]	28608318	1738	580	28608125	1841	614	28608124	1842	614	exon15_TKD1_beta1Sheet
-	19	rl_m4	double	no	21	77	0.011	0.00011	942046	104	False	[illegible]	[illegible]	28608276	1780	594	28608256	1800	600	28608255	1801	601	exon14_JMD_zipperMotif
-	19	rl_m4	double	no	36	49	0.133	0.00133	942046	1250	False	[illegible]	[illegible]	28608304	1752	584	28608269	1787	596	28608268	1788	596	exon14_JMD_zipperMotif
-	19	rl_m4	double	no	51	25	0.086	0.00086	942046	812	False	[illegible]	[illegible]	28608328	1728	576	28608278	1778	593	28608277	1779	593	exon14_JMD_zipperMotif
-	19	rl_m4	double	no	54	37	0.021	0.00021	942046	202	False	[illegible]	[illegible]	28608316	1740	580	28608263	1793	598	28608262	1794	598	exon14_JMD_zipperMotif
-	19	rl_m4	double	no	57	28	0.016	0.00016	942046	155	False	[illegible]	[illegible]	28608325	1731	577	28608269	1787	596	28608268	1788	596	exon14_JMD_zipperMotif
-	19	rl_m4	double	no	63	36	0.179	0.00179	942046	1685	False	[illegible]	[illegible]	28608317	1739	580	28608255	1801	601	28608254	1802	601	exon14_JMD_zipperMotif
-	19	rl_m4	double	no	93	38	17.484	0.21189	942046	164712	False	[illegible]	[illegible]	28608315	1741	581	28608223	1833	611	28608222	1834	612	exon14_TKD1_beta1Sheet
-	20	dx	double	no	30	42	0.074	0.00074	1238841	922	False	[illegible]	[illegible]	28608311	1745	582	28608282	1774	592	28608281	1775	592	exon14_JMD_switchMotif
-	20	dx	double	no	39	37	28.364	0.39595	1238841	351379	False	[illegible]	[illegible]	28608316	1740	580	28608278	1778	593	28608277	1779	593	exon14_JMD_zipperMotif
-	20	dx	double	no	48	20	0.198	0.00199	1238841	2458	False	[illegible]	[illegible]	28608333	1723	575	28608286	1770	590	28608285	1771	591	exon14_JMD_switchMotif
-	21	cr_cy2_c1	double	no	87	38	0.938	0.00947	2580998	24214	False	[illegible]	[illegible]	28608315	1741	581	28608229	1827	609	28608228	1828	610	exon14_TKD1_beta1Sheet
-	21	cr-fu-m3	double	no	87	38	0.061	0.00061	1076742	655	False	[illegible]	[illegible]	28608315	1741	581	28608229	1827	609	28608228	1828	610	exon14_TKD1_beta1Sheet
-	21	dx	double	no	21	68	0.101	0.00101	2246356	2261	False	[illegible]	[illegible]	28608285	1771	591	28608265	1791	597	28608264	1792	598	exon14_JMD_zipperMotif
-	21	dx	double	no	54	26	0.008	0.00008	2246356	180	False	[illegible]	[illegible]	28608327	1729	577	28608274	1782	594	28608273	1783	595	exon14_JMD_zipperMotif
-	21	dx	double	no	57	32	0.068	0.00068	2246356	1527	False	[illegible]	[illegible]	28608321	1735	579	28608265	1791	597	28608264	1792	598	exon14_JMD_zipperMotif
-	21	dx	double	no	87	38	38.089	0.61522	2246356	855612	False	[illegible]	[illegible]	28608315	1741	581	28608229	1827	609	28608228	1828	610	exon14_TKD1_beta1Sheet
-	21	rl_m5	double	no	87	38	35.688	0.55492	674936	240869	False	[illegible]	[illegible]	28608315	1741	581	28608229	1827	609	28608228	1828	610	exon14_TKD1_beta1Sheet
-	21	dx	single	no	21	68	0.029	0.00029	107012	31	False	[illegible]	[illegible]	28608285	1771	591	28608265	1791	597	28608264	1792	598	exon14_JMD_zipperMotif
-	21	dx	single	no	57	32	0.113	0.00113	107012	121	False	[illegible]	[illegible]	28608321	1735	579	28608265	1791	597	28608264	1792	598	exon14_JMD_zipperMotif
-	21	dx	single	no	87	38	34.724	0.53196	107012	37159	False	[illegible]	[illegible]	28608315	1741	581	28608229	1827	609	28608228	1828	610	exon14_TKD1_beta1Sheet
-	22	dx	double	no	39	93	46.136	0.85653	979941	452104	False	[illegible]	[illegible]	28608260	1796	599	28608222	1834	612	28608221	1835	612	exon14_TKD1_beta1Sheet
-	22	dx	double	no	78	54	0.008	0.00008	979941	79	False	[illegible]	[illegible]	28608299	1757	586	28608222	1834	612	28608221	1835	612	exon14_TKD1_beta1Sheet
-	22	dx	double	no	9	79	0.010	0.00010	115571	12	False	[illegible]	[illegible]	28608274	1782	594	28608266	1790	597	28608265	1791	597	exon14_JMD_zipperMotif
-	23	dx	double	no	54	43	41.156	0.69941	1411441	580889	False	[illegible]	[illegible]	28608310	1746	582	28608257	1799	600	28608256	1800	600	exon14_JMD_zipperMotif
-	23	dx	double	no	57	28	0.086	0.00086	1411441	1218	False	[illegible]	[illegible]	28608325	1731	577	28608269	1787	596	28608268	1788	596	exon14_JMD_zipperMotif
-	24	dx	double	no	66	61	39.744	0.65959	2135903	848885	False	[illegible]	[illegible]	28608292	1764	588	28608227	1829	610	28608226	1830	610	exon14_TKD1_beta1Sheet
-	25	cr_cy2_c1	double	no	21	82	0.053	0.00053	2565761	1354	False	[illegible]	[illegible]	28608271	1785	595	28608251	1805	602	28608250	1806	602	exon14_JMD_zipperMotif
-	25	cr_cy2_c1	double	no	36	41	0.061	0.00061	2565761	1561	False	[illegible]	[illegible]	28608312	1744	582	28608277	1779	593	28608276	1780	594	exon14_JMD_zipperMotif
-	25	cr_cy2_c1	double	no	39	40	0.050	0.00050	2565761	1295	False	[illegible]	[illegible]	28608313	1743	581	28608275	1781	594	28608274	1782	594	exon14_JMD_zipperMotif
-	25	cr_cy2_c1	double	no	42	25	0.048	0.00048	2565761	1236	False	[illegible]	[illegible]	28608328	1728	576	28608287	1769	590	28608286	1770	590	exon14_JMD_switchMotif
-	25	cr_cy2_c1	double	no	42	44	0.012	0.00012	2565761	319	False	[illegible]	[illegible]	28608309	1747	583	28608268	1788	596	28608267	1789	597	exon14_JMD_zipperMotif
-	25	cr_cy2_c1	double	no	60	31	2.004	0.02045	2565761	51428	False	[illegible]	[illegible]	28608322	1734	578	28608263	1793	598	28608262	1794	598	exon14_JMD_zipperMotif
-	25	cr-eot-c4	double	no	60	31	0.637	0.00642	1775585	11319	False	[illegible]	[illegible]	28608322	1734	578	28608263	1793	598	28608262	1794	598	exon14_JMD_zipperMotif
-	25	dx	double	no	21	82	0.718	0.00723	705386	5065	False	[illegible]	[illegible]	28608271	1785	595	28608251	1805	602	28608250	1806	602	exon14_JMD_zipperMotif
-	25	dx	double	no	24	54	0.008	0.00008	672529	53	False	[illegible]	[illegible]	28608299	1757	586	28608276	1780	594	28608275	1781	594	exon14_JMD_zipperMotif
-	25	dx	double	no	36	41	0.075	0.00075	672529	505	False	[illegible]	[illegible]	28608312	1744	582	28608277	1779	593	28608276	1780	594	exon14_JMD_zipperMotif
-	25	dx	double	no	39	40	0.367	0.00369	672529	2471	False	[illegible]	[illegible]	28608313	1743	581	28608275	1781	594	28608274	1782	594	exon14_JMD_zipperMotif
-	25	dx	double	no	42	25	15.830	0.18807	672529	106458	False	[illegible]	[illegible]	28608328	1728	576	28608287	1769	590	28608286	1770	590	exon14_JMD_switchMotif
-	25	dx	double	no	60	31	12.073	0.13731	672529	81192	False	[illegible]	[illegible]	28608322	1734	578	28608263	1793	598	28608262	1794	598	exon14_JMD_zipperMotif
-	25	dx	double	no	93	39	0.012	0.00012	672529	78	False	[illegible]	[illegible]	28608314	1742	581	28608222	1834	612	28608221	1835	612	exon14_TKD1_beta1Sheet
-	25	rl_m1	double	no	42	44	0.079	0.00079	1326595	1042	False	[illegible]	[illegible]	28608309	1747	583	28608268	1788	596	28608267	1789	597	exon14_JMD_zipperMotif
-	25	rl_m1	double	no	60	31	37.103	0.58990	1326595	492209	False	[illegible]	[illegible]	28608322	1734	578	28608263	1793	598	28608262	1794	598	exon14_JMD_zipperMotif
-	25	dx	single	no	21	82	0.610	0.00614	428573	2616	False	[illegible]	[illegible]	28608271	1785	595	28608251	1805	602	28608250	1806	602	exon14_JMD_zipperMotif
-	25	dx	single	no	24	54	0.010	0.00010	428573	41	False	[illegible]	[illegible]	28608299	1757	586	28608276	1780	594	28608275	1781	594	exon14_JMD_zipperMotif
-	25	dx	single	no	36	41	0.079	0.00079	428573	339	False	[illegible]	[illegible]	28608312	1744	582	28608277	1779	593	28608276	1780	594	exon14_JMD_zipperMotif
-	25	dx	single	no	36	56	0.006	0.00006	428573	26	False	[illegible]	[illegible]	28608297	1759	587	28608262	1794	598	28608261	1795	599	exon14_JMD_zipperMotif
-	25	dx	single	no	39	40	0.280	0.00281	428573	1200	False	[illegible]	[illegible]	28608313	1743	581	28608275	1781	594	28608274	1782	594	exon14_JMD_zipperMotif
-	25	dx	single	no	42	25	16.368	0.19571	428573	70149	False	[illegible]	[illegible]	28608328	1728	576	28608287	1769	590	28608286	1770	590	exon14_JMD_switchMotif
-	25	dx	single	no	60	31	12.205	0.13902	428573	52308	False	[illegible]	[illegible]	28608322	1734	578	28608263	1793	598	28608262	1794	598	exon14_JMD_zipperMotif
-	25	dx	single	no	93	39	0.007	0.00007	428573	31	False	[illegible]	[illegible]	28608314	1742	581	28608222	1834	612	28608221	1835	612	exon14_TKD1_beta1Sheet
-	26	dx	double	no	18	67	0.038	0.00038	1981236	756	False	[illegible]	[illegible]	28608286	1770	590	28608269	1787	596	28608268	1788	596	exon14_JMD_zipperMotif
-	26	dx	double	no	33	53	0.010	0.00010	1981236	207	False	[illegible]	[illegible]	28608300	1756	586	28608268	1788	596	28608267	1789	597	exon14_JMD_zipperMotif
-	26	dx	double	no	36	86	15.505	0.18350	1981236	307183	False	[illegible]	[illegible]	28608267	1789	597	28608232	1824	608	28608231	1825	609	exon14_JMD_hingeRegion
-	27	dx	double	no	72	40	0.011	0.00011	1648051	185	False	[illegible]	[illegible]	28608313	1743	581	28608242	1814	605	28608241	1815	605	exon14_JMD_hingeRegion
-	27	dx	double	no	96	39	48.290	0.93386	1648051	795848	False	[illegible]	[illegible]	28608314	1742	581	28608219	1837	613	28608218	-	-	intron14_spliceDonor
-	28	dx	double	no	197	32	9.946	0.11045	942898	93784	True	[illegible]	[illegible]	28608321	1735	579	28608125	1841	614	28608124	1842	614	exon15_TKD1_beta1Sheet
-	28	dx	double	no	36	64	0.047	0.00047	946585	441	False	[illegible]	[illegible]	28608289	1767	589	28608254	1802	601	28608253	1803	601	exon14_JMD_zipperMotif
-	28	dx	double	no	57	72	0.007	0.00007	946585	68	False	[illegible]	[illegible]	28608281	1775	592	28608225	1831	611	28608224	1832	611	exon14_TKD1_beta1Sheet
-	28	dx	double	no	60	70	0.038	0.00038	946585	355	False	[illegible]	[illegible]	28608283	1773	591	28608224	1832	611	28608223	1833	611	exon14_TKD1_beta1Sheet
-	28	dx	double	no	75	50	0.029	0.00029	946585	277	False	[illegible]	[illegible]	28608303	1753	585	28608229	1827	609	28608228	1828	610	exon14_TKD1_beta1Sheet
-	28	dx	double	no	84	51	0.259	0.00260	946585	2452	False	[illegible]	[illegible]	28608302	1754	585	28608219	1837	613	28608218	-	-	intron14_spliceDonor
-	28	rl_m4	double	no	197	32	0.056	0.00056	1953484	1086	True	[illegible]	[illegible]	28608321	1735	579	28608125	1841	614	28608124	1842	614	exon15_TKD1_beta1Sheet
-	28	dx	single	no	18	73	0.014	0.00014	154739	21	False	[illegible]	[illegible]	28608280	1776	592	28608263	1793	598	28608262	1794	598	exon14_JMD_zipperMotif
-	28	dx	single	no	197	32	20.593	0.25933	154196	31753	True	[illegible]	[illegible]	28608321	1735	579	28608125	1841	614	28608124	1842	614	exon15_TKD1_beta1Sheet
-	28	dx	single	no	33	41	0.010	0.00010	154739	16	False	[illegible]	[illegible]	28608312	1744	582	28608280	1776	592	28608279	1777	593	exon14_JMD_zipperMotif
-	28	dx	single	no	36	64	0.062	0.00062	154739	96	False	[illegible]	[illegible]	28608289	1767	589	28608254	1802	601	28608253	1803	601	exon14_JMD_zipperMotif
-	28	dx	single	no	57	72	0.017	0.00017	154739	27	False	[illegible]	[illegible]	28608281	1775	592	28608225	1831	611	28608224	1832	611	exon14_TKD1_beta1Sheet
-	28	dx	single	no	60	70	0.067	0.00067	154739	104	False	[illegible]	[illegible]	28608283	1773	591	28608224	1832	611	28608223	1833	611	exon14_TKD1_beta1Sheet
-	28	dx	single	no	75	50	0.006	0.00006	154739	10	False	[illegible]	[illegible]	28608303	1753	585	28608229	1827	609	28608228	1828	610	exon14_TKD1_beta1Sheet
-	28	dx	single	no	84	51	0.185	0.00185	154739	286	False	[illegible]	[illegible]	28608302	1754	585	28608219	1837	613	28608218	-	-	intron14_spliceDonor
10^-0	MOLM-14	-	double	no	21	72	64.979	1.85540	1608216	1045001	FALSE	[illegible]	[illegible]	28608281	1775	592	28608261	1795	599	28608260	1796	599	exon14_JMD_zipperMotif
10^-1	MOLM-14	-	double	no	21	72	8.638	0.09454	2534728	218942	False	[illegible]	[illegible]	28608281	1775	592	28608261	1795	599	28608260	1796	599	exon14_JMD_zipperMotif
10^-2	MOLM-14	-	double	no	21	72	0.880	0.00888	2559104	22531	False	[illegible]	[illegible]	28608281	1775	592	28608261	1795	599	28608260	1796	599	exon14_JMD_zipperMotif
10^-2	MOLM-14	-	double	no	21	72	0.721	0.00726	1597234	11511	FALSE	[illegible]	[illegible]	28608281	1775	592	28608261	1795	599	28608260	1796	599	exon14_JMD_zipperMotif
10^-2	MOLM-14	-	double	no	21	72	0.727	0.00732	1542260	11205	FALSE	[illegible]	[illegible]	28608281	1775	592	28608261	1795	599	28608260	1796	599	exon14_JMD_zipperMotif
10^-3	MOLM-14	-	double	no	21	72	0.085	0.00086	2721929	2326	False	[illegible]	[illegible]	28608281	1775	592	28608261	1795	599	28608260	1796	599	exon14_JMD_zipperMotif
10^-3	MOLM-14	-	double	no	21	72	0.075	0.00075	1182257	885	FALSE	[illegible]	[illegible]	28608281	1775	592	28608261	1795	599	28608260	1796	599	exon14_JMD_zipperMotif
10^-3	MOLM-14	-	double	no	21	72	0.060	0.00060	1070962	639	FALSE	[illegible]	[illegible]	28608281	1775	592	28608261	1795	599	28608260	1796	599	exon14_JMD_zipperMotif
10^-4	MOLM-14	-	double	no	21	72	0.005	0.00005	2295906	111	False	[illegible]	[illegible]	28608281	1775	592	28608261	1795	599	28608260	1796	599	exon14_JMD_zipperMotif
10^-4	MOLM-14	-	double	no	21	72	0.015	0.00015	1517828	229	FALSE	[illegible]	[illegible]	28608281	1775	592	28608261	1795	599	28608260	1796	599	exon14_JMD_zipperMotif
10^-4	MOLM-14	-	double	no	21	72	0.001	0.00001	1641666	12	FALSE	[illegible]	[illegible]	28608281	1775	592	28608261	1795	599	28608260	1796	599	exon14_JMD_zipperMotif
10^-4	MOLM-14	-	double	no	21	72	0.001	0.00001	1473097	11	FALSE	[illegible]	[illegible]	28608281	1775	592	28608261	1795	599	28608260	1796	599	exon14_JMD_zipperMotif
10^-0	PL-21	-	double	no	125	24	18.641	0.22912	2019586	376467	True	[illegible]	[illegible]	28608329	1727	576	28608205	-	-	28608204	-	-	intron14
10^-1	PL-21	-	double	no	125	24	2.623	0.02693	2230257	58495	True	[illegible]	[illegible]	28608329	1727	576	28608205	-	-	28608204	-	-	intron14
10^-2	PL-21	-	double	no	125	24	0.260	0.00261	2101957	5465	True	[illegible]	[illegible]	28608329	1727	576	28608205	-	-	28608204	-	-	intron14
10^-3	PL-21	-	double	no	125	24	0.015	0.00015	1361576	204	True	[illegible]	[illegible]	28608329	1727	576	28608205	-	-	28608204	-	-	intron14

Table S3: *getITD* results: Shown are the detailed results obtained by *getITD* for each *FLT3*-ITD that was detected by our assay in the samples listed in Table S1. Genomic coordinates are based on hg19, transcriptomic and proteomic coordinates refer to NCBI `NM_004119`.

Patient ID	Age at dx (years)	Sex	Type of AML	Time from dx to rl (days)	CR after induction	BM blasts at dx (%)	PB blasts at dx (%)	BM blasts at rl (%)	PB blasts at rl (%)	*NPM1*-mutated at dx	*NPM1* transcripts at dx (in BM)	*NPM1* transcripts at rl (in BM)	Induction cycles (Dauna-ARAC 7+3)	Consolidation cycles (High dose ARAC)	Transplanted in 1st CR	WBC at dx (G/l)	WBC at rl (G/l)
13	65.15	male	de novo	561.00	CRi	95	98	5	0	yes	104882	22022	1	4	no	166.6	5.2
19	25.03	male	de novo	182.00	CR	69	11	32	na	no	na	na	2	0	yes	10.7	na
21	52.14	male	de novo	325.00	CRi	90	59	43	4	no	na	na	2	0	yes	na	2.3
25	53.49	male	de novo	245.00	CR	80	37	90	25	yes	537470	3682990	1	4	no	na	6.6
28	51.52	male	de novo	289.00	CRi	80	29	na	8	no	na	na	1	3	no	52.8	3.5

Table S4: Clinical data: Provided are clinical details of five *FLT3*-ITD positive AML patients that relapsed during follow-up (Figure 2A).
